# *Schistosoma japonicum* infection causes a reprogramming of glycolipid metabolism in the liver

**DOI:** 10.1186/s13071-019-3621-6

**Published:** 2019-08-02

**Authors:** Zhi-Peng Xu, Hao Chang, Yang-Yue Ni, Chen Li, Lin Chen, Min Hou, Min-Jun Ji

**Affiliations:** 1 Department of Pathogen Biology, Nanjing Medical University, Nanjing, 211166 Jiangsu People’s Republic of China; 2 Jiangsu Province Key Laboratory of Modern Pathogen Biology, Nanjing Medical University, Nanjing, 211166 Jiangsu People’s Republic of China

**Keywords:** *Schistosoma japonicum*, Macrophages, Metabolism

## Abstract

**Background:**

Recent investigations indicate that schistosome infection is closely associated with aberrant glycolipid metabolism. However, the actual glycolipid metabolism gene expression, as well as the possible pathways that regulate glycolipid metabolism in the schistosome-infected liver, has not been extensively explored.

**Methods:**

In this study, we evaluated the dynamic expression of glycolipid metabolism-associated genes and proteins in the livers from mice infected with *Schistosoma japonicum* at the indicated time points using real-time PCR and immunofluorescence. Then, cultures of macrophages were treated with schistosome soluble egg antigen (SEA) to detect the expression levels of genes associated with glucose and lipid metabolism in order to identify macrophages metabolic characteristics in response to these antigens. Furthermore, SEA-stimulated macrophages were co-cultures with hepatocytes and detected the effects of macrophages on the gene expression of hepatocytes metabolism.

**Results:**

The expression of glycolysis-related genes (Ldha, Glut4, Pkm2, Glut1, Pfkfb3, Aldoc, HK2, Pfk) in the liver were upregulated but the gluconeogenesis gene (G6pc) was downregulated during *S. japonicum* infection. In addition, the mRNA levels of fatty acid (FA) oxidation-related genes (Ucp2, Atp5b, Pparg) in the liver were significantly upregulated; however, the FA synthesis genes (Fas, Acc, Scd1, Srebp1c) and lipid uptake gene (Cd36) were downregulated post*-S. japonicum*-infection. In consistence with these data, stimulation with SEA *in vitro* significantly enhanced the gene expression that involved in glycolysis and FA oxidation, but decreased genes related to gluconeogenesis, FA synthesis and lipid uptake in macrophages. The levels of phosphorylated AMPK, AKT and mTORC1 were increased in macrophages after SEA stimulation. Inhibition of phosphorylated AMPK, AKT and mTORC1 promoted SEA-treated macrophages to produce glucose. In addition, suppression of phosphorylated-AMPK, but not phosphorylated-AKT and phosphorylated-mTOR, induced the lipid accumulation in SEA-stimulated macrophages. Furthermore, SEA-treated macrophages significantly reduced the expression of Acc mRNA in hepatocytes *in vitro*.

**Conclusions:**

These findings reveal *S. japonicum* infection induces dynamic changes in the expression levels of genes involved in catabolism (glucose uptake, glycolysis and fatty acid oxidation) and suppressing anabolism (glycogen synthesis) in the liver, which could occur *via* macrophages’ metabolic states, particularly those involved in the AMPK, AKT and mTORC1 pathways.

**Electronic supplementary material:**

The online version of this article (10.1186/s13071-019-3621-6) contains supplementary material, which is available to authorized users.

## Background

Recently, increasing attention has focused on the inverse association between schistosome infection, the most chronic helminth infections in humans in nature, and metabolism-related diseases, including obesity and diabetes [1, 2]. Recently, several studies have suggested that specific immune responses, such as anti-inflammatory response, eosinophilia and M2 polarization, might contribute at least partly to this issue [3–5]. Importantly, our previous study indicated that chronic *S. japonicum* infection or SEA treatment in the mouse model can regulate host metabolic homeostasis through promoting Th2 responses in the liver [5]. However, the potential mechanism that regulates the glucose and lipids metabolism of the liver during *S. japonicum* infection remains unclear.

The liver, a main metabolic organ of the body, plays major metabolic roles in regulating the homeostasis of glucose and lipids, as well as amino acids during alterations of metabolic conditions [6]. As one of the major cellular constituents and important immune regulators of liver pathology in schistosomiasis [7–9], macrophages have been reported to play critical roles in maintaining hepatic informatory and metabolic homeostasis. In response to environmental signals, macrophages undergo polarized activation to classically activated macrophages (M1) or alternatively activated macrophages (M2)-like activation states. The M1 macrophages exhibit a high level of arginine metabolism and maintain the glycolytic activity by enhancing the expression of the pro-glycolytic 6-phosphofructo-2-kinase/fructose-2,6-bisphosphatase-3 (PFKFB3); M2 macrophages shift macrophage metabolism into fatty acid oxidation (FAO) and oxidative phosphorylation (OXPHOS) states, directed by signaling *via* IL-4 [10]. It has been proposed that during weight gain macrophages undergo a “switch” from an anti-inflammatory M2 to a pro-inflammatory M1 state, that may contribute to systemic insulin resistance, the anti-inflammatory cytokine IL-10, which is produced from M2 macrophages, protects adipocytes from TNF-α-induced insulin resistance [11]. Interestingly, injections with *S. mansoni*-soluble egg antigens (SEA) improved peripheral glucose uptake and high-fat-diet-induced insulin resistance and obesity, which was associated with the increased adipose tissue M2 macrophages [12]. PF4178903, dual CCR2/CCR5 antagonism, has been reported to limit the adipocyte size and inhibit body weight through inducing a shift into the M2-dominant phenotype [13]. Therefore, it is suggested that maintaining the balance of macrophage metabolic homeostasis may be a potential therapeutic strategy for metabolic diseases.

The dynamics of liver metabolism during schistosome infection have not yet been investigated. In addition, the possible effects of schistosomal egg antigens on macrophages metabolism are still unknown. Our study systematically investigated the kinetics of the gene expression related to hepatic glycolipid metabolism in experimentally *S. japonicum*-infected mice and examined the potential effects of schistosomal egg antigens on the macrophage metabolism, which provide a better understanding of the mechanisms by which helminth infection regulates host metabolic homeostasis.

## Methods

### Mice

Six-week-old female C57BL/6 mice purchased from the Animal Core Facility of Nanjing Medical University were bred under specific-pathogen-free (SPF) conditions.

### *Schistosoma japonicum* infection and preparation of antigen

Each mouse was infected with 12 cercariae of *S. japonicum*. Six mice were randomly chosen from the infected and normal control groups at 0, 4, 7 and 11 weeks post-infection in each independent experiment, and all the mice were euthanized *via* diethyl ether-induced anesthesia for further study.

Schistosome egg antigen (SEA) was obtained from Jiangsu Institute of Parasitic Diseases, Wuxi, China, and prepared following a previously described method with some modifications [14]. Briefly, rabbits were infected by ~ 2000 cercariae, and their livers were collected and cut into a number of pieces six to seven weeks post-infection. Then, the liver pieces were homogenized in PBS on ice and then filtered, washed and centrifuged. Purified eggs in pre-cooled PBS were sonicated three times for 15 min. The suspension was frozen/thawed several times and centrifuged at 25,000×*g* for 30 min. A 0.22-μm filter was used to pass the supernatant and obtain SEA, which was evaluated for contaminating endotoxin (LPS) by the chromogenic LAL end-point assay kit (Cambrex, Charles City, IO, USA). The concentration of LPS in SEA was below 1.2 endotoxin units (EU)/mg. A bicinchoninic acid (BCA) Protein Assay kit (Bio-Rad, Richmond, CA, USA) was used to determine the protein concentration.

### Fasting blood glucose detection

Blood was collected from the tail of fasting control and *S. japonicum-*infected mice (from 6:00 to 10:00 h), and then the fasting blood glucose (FBG) was measured using a hand-held glucometer (Bayer Breeze 2; Bayer Inc., Toronto, ON, Canada).

### Cell lines and *in vitro* treatment

RAW264.7 cells were purchased from the Cell Bank of Chinese Academy of Sciences. RAW264.7 cells were cultured on plastic aseptic dishes in DMEM, supplemented with 10% FBS, and penicillin (100 IU/ml)/streptomycin (100 mg/ml). Cells were maintained at 37 °C, 5% CO_2_ in a humidified atmosphere.

For antigen treatment, PBS (control) or 20 μg/ml SEA were added directly to each well and then incubated for 24 h at 37 °C.

### Quantitative RT-PCR

RNA from liver tissue and cells was extracted with a RNeasy Mini Kit (Qiagen, Hilden, Germany). Then, RNA was reverse-transcribed by using a RevertAid™ First Strand cDNA Synthesis Kit (Fermantas Life Sciences, St. Leon-Rot, Baden-Württemberg, Germany). Real-time PCR was performed using Power Syber Green PCR Master Mix (Applied Biosystems, Foster City, CA, USA). The housekeeping gene β-actin was used to normalize the gene expression by using the 2^−ΔΔCt^ method. All primer sequences are shown in Additional file 1: Table S1.

### Western blotting

An equal amount of protein (~ 30 μg) in each group was electrophoresed by SDS-PAGE and were then was then blotted onto nitrocellulose. Then, the membranes were blocked in 5% milk for 2 h, washed and incubated with primary antibodies: rabbit anti-phospho-ACC (Abcam, Cambridge, MA, USA), rabbit anti-ACC (Abcam), rabbit anti-phospho-AKT (Bioworld Technology, Louis Park, MN, USA), rabbit anti-AKT (Bioworld), rabbit anti-phospho-AMPKɑ (Cell Signaling Technology, Beverly, MA, USA), rabbit anti-AMPKɑ (D5A2) (Cell Signaling Technology), anti-phospho-mTORC1 (Bioworld), rabbit anti-mTORC1(S2442) (Bioworld), rabbit anti-phospho-PI3K (Bioworld), rabbit anti-PI3K P85α (Bioworld), or rabbit anti-β-actin (Cell Signaling Technology). Then, membranes were incubated for 1 h with HRP-conjugated anti-rabbit IgG antibody (Cell Signaling Technology). Then, the blots were developed using the ECL system (GE Healthcare, Little Chalfont, UK) and band density was quantified by Image J software (Image Processing and Analysis in Java, National Institutes of Health, Bethesda, MD, USA) and normalized to β-actin.

### Immunofluorescence

Liver tissue from each group was fixed with 4% paraformaldehyde and permeabilized with 0.5% Triton X-100 in PBS for 15 min at room temperature. Unspecific binding sites were blocked with 2% BSA for 30 min at 4 °C. Slides were stained with rabbit anti-phospho-AKT (Bioworld). After staining, slides were washed with PBS and Alexa Fluor 488-conjugated goat anti-rabbit IgG antibody (1:1000 dilution) was added. Finally, samples were washed then analyzed under a microscope by using AxioVision Rel 4.7 (Carl Zeiss GmbH, Jena, Germany). Quantification of fluorescence was achieved by using Image-ProPlus software v.6.0 (Media Cybernetics, Silver Spring, MD, USA).

### Measurements of glucose levels

A total of 1 × 10^6^ RAW264.7 cells were seeded into 12-well plates and the medium was changed after 6 h. Cells were stimulated with SEA (25 μg/ml) with or without the inhibitors of p-AMPK (dorsomorphin, MEC, 10 μmol), p-AKT (dihydrochloride, 10 μmol) or p-mTOR (rapamycin, 5 μmol) for 24 h, then the culture medium was collected for measurement of glucose concentrations. Glucose levels were determined using a Glucose assay kit (Sigma-Aldrich, St. Louis, MO, USA).

### Oil red O staining

Intracellular lipid accumulation of RAW264.7 cells was determined by oil red O staining. Cells were fixed with 4% formaldehyde for 15 min at room temperature and stained with oil red O solution (0.5% oil red O in isopropyl alcohol/water = 3:2) for 2 h. After staining, the cells were washed three times with water to remove excess stain. The quantification of intracellular lipid by oil red O staining (lipid droplets positive cells *vs* the cell section area) was performed using Image J software.

### Coculture conditions

A total of 1 × 10^6^ RAW264.7 cells were separated into 12-well plates and stimulated with SEA (25 μg/ml) for 24 h at 37 °C. Then, unstimulated FL83b cells (1 × 10^6^) were seeded in the lower chamber of the transwell, and the SEA-activated RAW264.7 cells (1 × 10^6^) were added into the upper chamber and co-cultured for another 24 h. After that, the FL83b cells were collected in the lower chamber and the expression of metabolic genes using real-time PCR was analyzed.

### Statistical analysis

All analyses were carried out with SPSS v.19.0 software. Data are shown as the mean ± standard deviation (SD). The significance of the difference between two groups was identified using Student’s t-test. Multiple comparisons were performed by one-way ANOVA and followed by LSD *post-hoc* test for comparison between every two groups. *P*-values < 0.05 were considered significant. Graphs were generated using the software GraphPad Prism v.7.0.

## Results

### Expression profiling of hepatic genes associated with glucose metabolism during *Schistosoma japonicum* infection

**Fig. 1:**
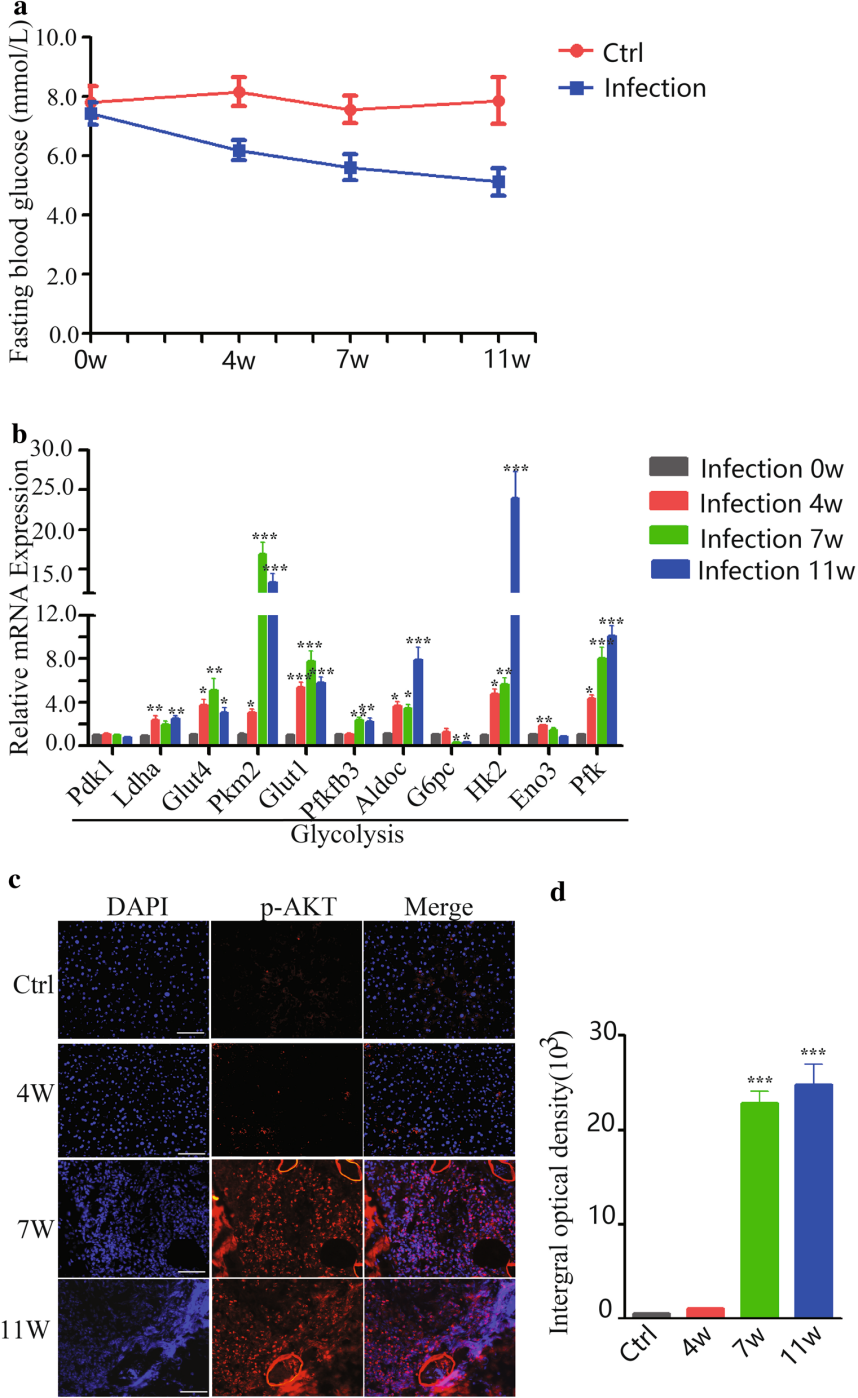
Dynamic expression profiling of glucose metabolism-related genes in the liver of *S. japonicum-*infected mice. **a** Fasting blood glucose was detected from normal or *S. japonicum*-infected mice at indicated time points (0, 4, 7 and 11 weeks post-infection), and analyzed using two-way ANOVA. **b** Liver tissues were prepared from *S. japonicum*-infected mice at indicated time points (0, 4, 7 and 11 weeks post-infection). The mRNA levels of glycolysis-related important genes (Pdk1, Ldha, Glut4, Pkm2, Glut1, Pfkfb3, Aldoc, HK2, Eno3, Pfk) and gluconeogenesis gene (G6pc) in the liver of each group were evaluated by real-time PCR. The mRNA level of each gene was normalized to β-actin mRNA levels in each sample. **c**, **d** Immunofluorescence of Pho-AKT in the liver tissue from normal or *S. japonicum*-infected mice (0, 4, 7 and 11 weeks post-infection); original magnification, ×200. The mean optical density of Pho-AKT positive cells from 10 random fields in each mouse was digitized and analyzed using Image-Pro Plus software. Data are expressed as the mean ± SD of three independent experiments with 6 mice per group in each experiment (ANOVA/LSD: *P < 0.05, **P < 0.01, ***P < 0.001). *Scale-bars*: **c**, **d**, 50 μm

Our previous study showed that the whole-body and hepatic insulin sensitivity were decreased in *S. japonicum*-infected mice [5]. In consistence with this, the level of fasting blood glucose in mice was significantly decreased after *S. japonicum* infection (Fig. 1a; two-way ANOVA; *F*_(1, 5)_ = 11.68, *P* = 0.0066). To investigate the specific glucose metabolism-related genes involved in this process, we profiled the dynamic mRNA expression of glucose metabolic genes in the livers of *S. japonicum* infected mice. As shown in Fig. 1b, the kinetics of glycolysis-related genes expression (Ldha, Glut4, Pkm2, Glut1, Pfkfb3, Aldoc, Hk2, Pfk) (ANOVA and LSD multiple comparison test; Ldha: *F*_(3, 23)_ = 6.511, *P* = 0.0030; Glut4: *F*_(3, 23)_ = 6.276, *P* = 0.0035; Pkm2: *F*_(3, 23)_ = 65.51, *P* < 0.0001; Glut1: *F*_(3, 23)_ = 22.53, *P* < 0.0001; Pfkfb3: *F*_(3, 23)_ = 9.281, *P* = 0.0005; Aldoc: *F*_(3, 23)_ = 19.09, *P* < 0.0001; Hk2: *F*_(3, 23)_ = 34.54, *P* < 0.0001; Eno3: *F*_(3, 23)_ = 14.39, *P* < 0.0001; Pfk: *F*_(3, 23)_ = 28.85, *P* < 0.0001) in the liver were upregulated but G6pc, a gluconeogenesis gene, was downregulated after *S. japonicum* eggs were deposited in the liver (ANOVA and LSD multiple comparison test; G6pc: *F*_(3, 23)_ = 8.217, *P* = 0.009). The above genes were not changed in control mice at different time points corresponding to infected mice (Additional file 2: Figure S1; ANOVA and LSD multiple comparison test; pdk1: *F*_(3, 23)_ = 0.062, *P* = 0.9790; Ldha: *F*_(3, 23)_ = 1.375, *P* = 0.2791; Glut4: *F*_(3, 23)_ = 0.0796, *P* = 0.9706; Pkm2: *F*_(3, 23)_ = 0.373, *P* = 0.7731; Glut1: *F*_(3, 23)_ = 0.161, *P* = 0.9214; Pfkfb3: *F*_(3, 23)_ = 0.8802, *P* = 0.4680; Aldoc: *F*_(3, 23)_ = 0.895, *P* = 0.4610; G6pc: *F*_(3, 23)_ = 0.687, *P* = 0.5706; Hk2: *F*_(3, 23)_ = 0.183, *P* = 0.9069; Eno3: *F*_(3, 23)_ = 1.234, *P* = 0.3235; Pfk: *F*_(3, 23)_ = 0.202, *P* = 0.8940). Since serine/threonine kinase AKT is the key factor that directly influences glucose metabolism [15], we next detected the level of hepatic phosphorylated AKT (T308) in mice with *S. japonicum* infection by using immunofluorescence. As expected, the expression of hepatic phosphorylated AKT was significantly enhanced in the liver at 7 and 11 weeks post-infection (Fig. 1c, d). Together, these results indicate that *S. japonicum* infection promotes metabolic gene expression of hepatic glycolysis, but inhibits gene expression related to glucose synthesis.

### Dynamics of hepatic lipid metabolic genes during *Schistosoma japonicum* infection

**Fig. 2:**
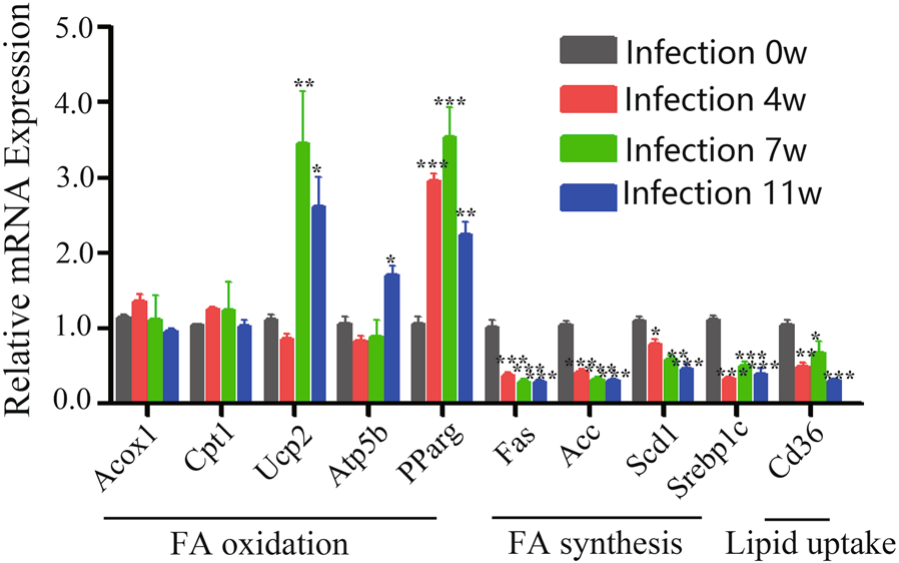
Dynamic of the expression of genes associated with hepatic lipid metabolism during *S. japonicum* infection. Liver tissues were prepared from *S. japonicum* infected mice at 0, 4, 7 and 11 weeks post-infection. The expression of genes related to fatty acid (FA) oxidation (Acox1, Cpt1, Ucp2, Atp5b, Pparg), FA synthesis (Fas, Acc, Scd1, Srebp1c) and lipid uptake (CD36) in the liver was evaluated by real-time PCR. The mRNA level of each gene was normalized to β-actin mRNA levels in each sample (ANOVA/LSD: *P < 0.05, **P < 0.01, ***P < 0.001)

Our unpublished data indicated that the serum cholesterol and triglycerides levels in mice were decreased post-*S. japonicum*-infection. We next assessed the kinetics of lipid metabolism-related genes expression during *S. japonicum* infection (Fig. 2). Results showed that the hepatic mRNA expression of fatty acid (FA) oxidation-related genes (Ucp2, Atp5b, Pparg) (ANOVA and LSD multiple comparison test; Ucp2: *F*_(3, 23)_ = 8.761, *P* = 0.0007; Atp5b: *F*_(3, 23)_ = 7.939, *P* = 0.0011; Pparg: *F*_(3, 23)_ = 20.97, *P* < 0.0001) in *S. japonicum* infected mice were significantly upregulated. However, the FA synthesis genes (Fas, Acc, Scd1, Srebp1c) (ANOVA; Fas: *F*_(3, 23)_ = 37.56, *P* < 0.0001; Acc: *F*_(3, 23)_ = 54.63, *P* < 0.0001; Scd1: *F*_(3, 23)_ = 19.08, *P* < 0.0001; Srebp1c: *F*_(3, 23)_ = 33.57, *P*  < 0.0001) and lipid uptake gene (CD36) (ANOVA and LSD multiple comparison test; CD36: *F*_(3, 23)_ = 12.10, *P* < 0.0001) were downregulated after *S. japonicum* egg deposition (Fig. 2b). These lipid metabolism-related genes were not changed in control mice at the time points corresponding to infected mice (Additional file 3: Figure S2; ANOVA and LSD multiple comparison test; Acox1: *F*_(3, 23)_ = 1.114, *P* = 0.3668; cpt1: *F*_(3, 23)_ = 0.6700, *P* = 0.5804; Ucp2: *F*_(3, 23)_ = 0.2035, *P* = 0.8928; Atp5b: *F*_(3, 23)_ = 0.2363, *P* = 0.8700; Pparg: *F*_(3, 23)_ = 0.9792, *P* = 0.4223; Fas: *F*_(3, 23)_ = 0.3470, *P* = 0.7917; Acc: *F*_(3, 23)_ = 0.2448, *P* = 0.8641; Scd1: *F*_(3, 23)_ = 1.276, *P* = 0.3096; Srebp1c: *F*_(3, 23)_ = 0.3687, *P* = 0.7764; CD36: *F*_(3, 23)_ = 0.5118, *P* = 0.6788) (Fig. 2a).

### The effects of SEA on macrophage glycolipid metabolism *in vitro*

**Fig. 3:**
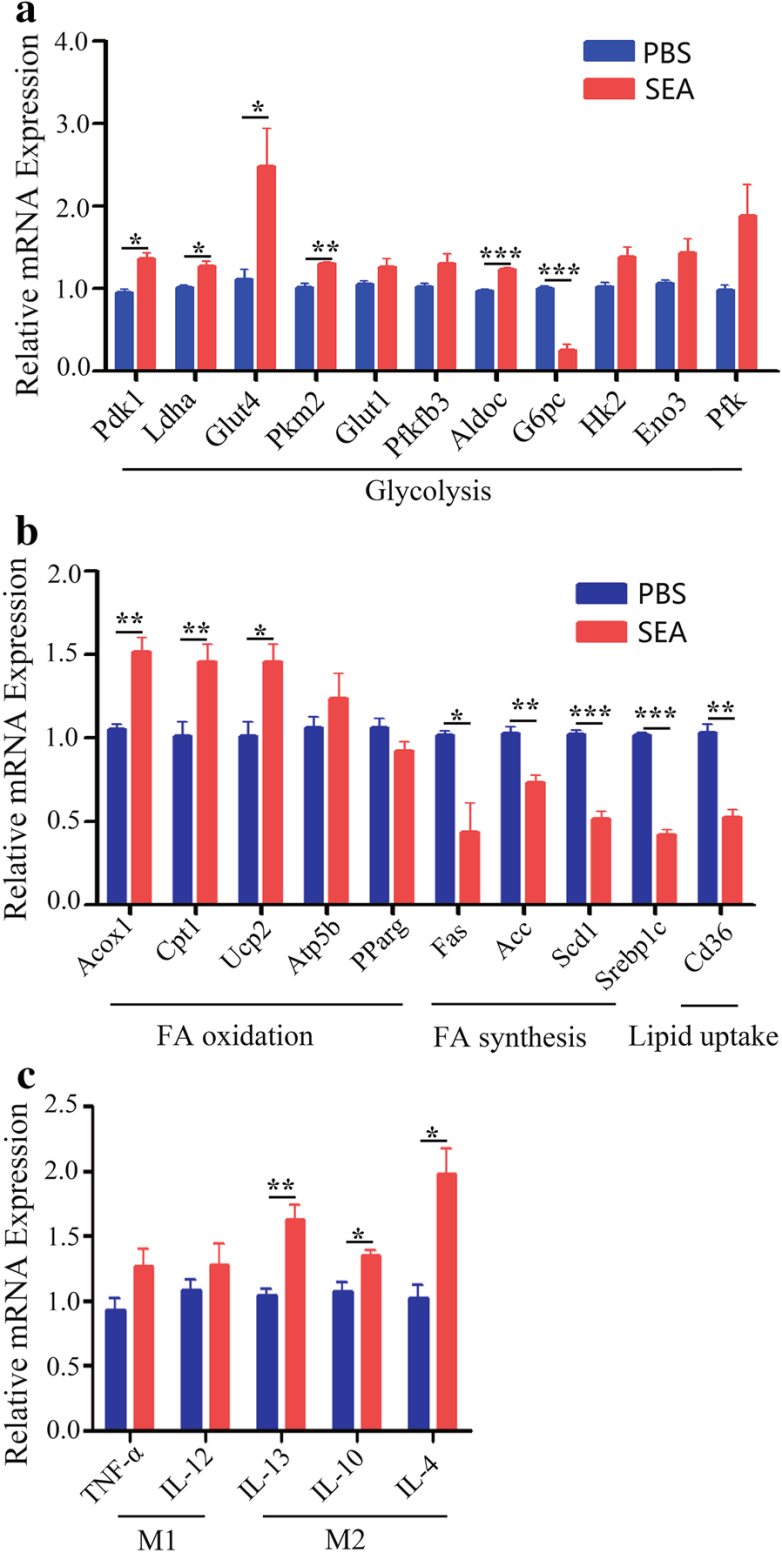
The effects of SEA on the expression of genes associated with glycolipid metabolism in macrophage *in vitro*. PBS (control) or 20 μg/ml SEA was added into RAW264.7 cells for 24 h at 37 °C. The expression of genes related glucose metabolism (**a**), lipid metabolism (**b**) and inflammatory factors (**c**) in RAW264.7 cells was evaluated by real-time PCR. The expression levels for each gene were normalized to the level in macrophages treated with PBS. Data are expressed as the mean ± SD of three independent experiments with similar results (Student’s t-test: *P < 0.05, **P < 0.01, ***P < 0.001)

Since macrophages have been shown to play important roles in the metabolic functions in the liver [16], we next investigated whether SEA could induce changes in macrophage energy metabolism *in vitro*. As shown in Fig. 3a, stimulation with SEA significantly enhanced the gene expression involved in glycolysis (Pdk1, Ldha, Glut4, Pkm2, Aldoc) (t-test; Pdk1: *t*_(4)_ = 4.387, *P* = 0.0118; Ldha: *t*_(4)_ = 3.890, *P* = 0.0177; Glut4: *t*_(4)_ = 2.943, *P* = 0.0423; Pkm2: *t*_(4)_ = 5.129, *P* = 0.0068; Glut1: *t*_(4)_ = 1.979, *P* = 0.1189; Pfkfb3: *t*_(4)_ = 2.242, *P* = 0.0884; Aldoc: *t*_(4)_ = 9.410, *P* = 0.0007; HK2: *t*_(4)_ = 2.775, *P* = 0.0501; Eno3: *t*_(4)_ = 2.049, *P* = 0.1099; Pfk: *t*_(4)_ = 2.330, *P* = 0.0803). However, the expression of G6pc mRNA was decreased after SEA stimulation in macrophages (t-test: *t*_(4)_ = 9.149, *P* = 0.0008). In addition, stimulation with SEA resulted in upregulation of FA oxidation-related genes in macrophages, as evidenced by significantly increased expression of Acox1, Cpt1 and Ucp2 (Fig. 3b) (t-test; Acox1: *t*_(4)_ = 5.145, *P* = 0.0068; Cpt1: *t*_(4)_ = 3.314, *P* = 0.0295; Ucp2: *t*_(4)_ = 3.311, *P* = 0.0296; Atp5b: *t*_(4)_ = 1.040, *P* = 0.3571; Pparg: *t*_(4)_ = 1.888, *P* = 0.1320). Furthermore, SEA also inhibited FA synthesis and lipid uptake in macrophages, as evidenced by markedly decreased gene expression of Fas, Acc, Scd1, Srebplc and Cd36 (Fig. 3b) (t-test; Fas: *t*_(4)_ = 3.294, *P* = 0.0301; Acc: *t*_(4)_ = 5.360, *P* = 0.0058; Scd1: *t*_(4)_ = 9.452, *P* = 0.0007; Srebpl: *t*_(4)_ = 16.98, *P* < 0.0001; Cd36: *t*_(4)_ = 7.190, *P* = 0.0020). A previous study showed that the oxidative metabolism in macrophages provided the energy for M2 macrophages polarization [17]. Consistently, SEA treated macrophages showed a M2-dominant phenotype, as evidenced by increased expression of IL-13, IL-10 and IL-4 (t-test; IL-13: *t*_(4)_ = 4.658, *P* = 0.0096; IL-10: *t*_(4)_ = 3.494, *P* = 0.0250; IL-4: *t*_(4)_ = 4.372, *P* = 0.0120), but not TNF-α and IL-12 (t-test; TNF-α: *t*_(4)_ = 1.952, *P* = 0.1084; IL-12: *t*_(4)_ = 1.037, *P* = 0.358) (Fig. 3c).

### Activation of metabolic sensors by SEA on macrophages

**Fig. 4:**
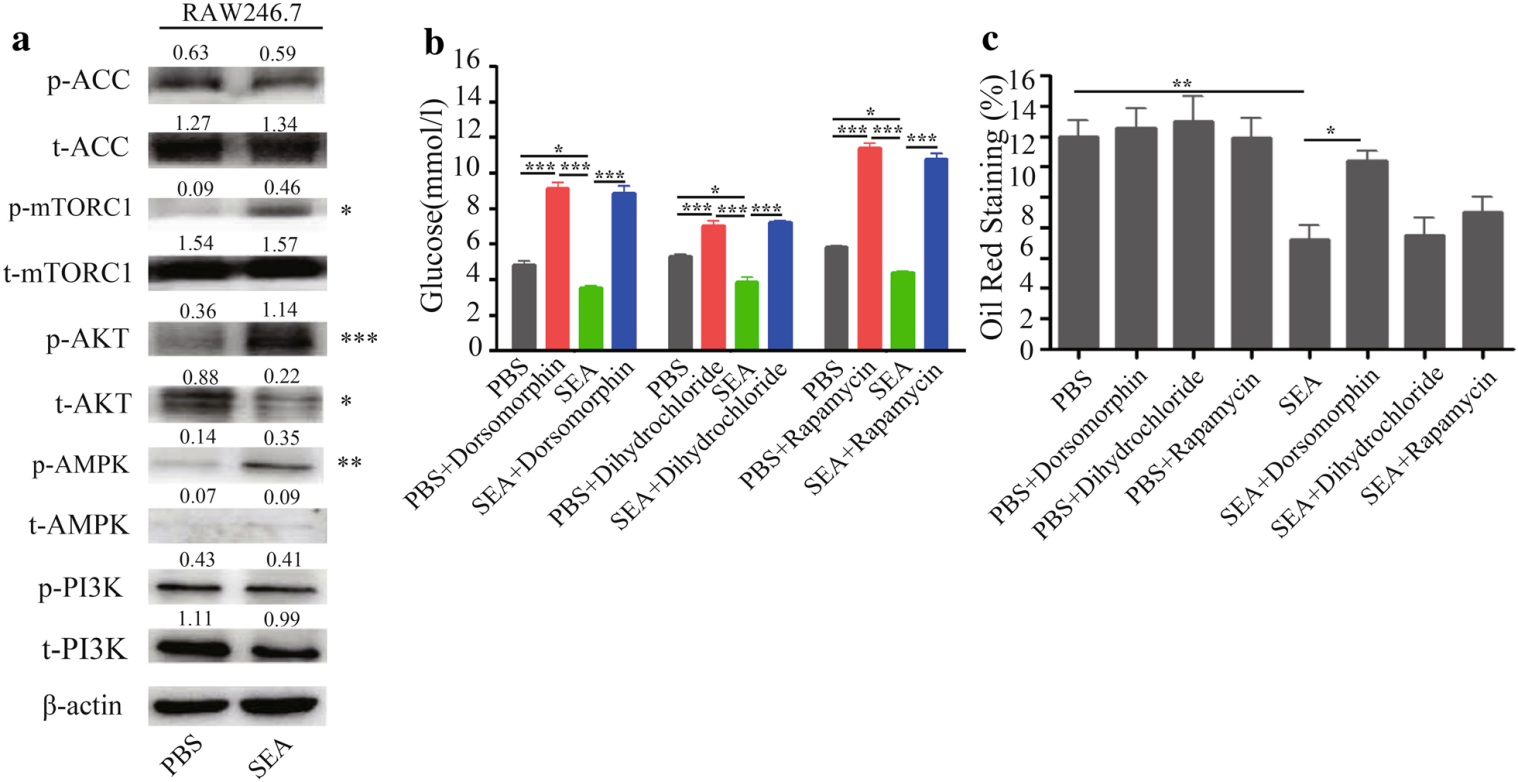
Effect of SEA on the key metabolic signaling molecular in macrophage *in vitro*. **a** Immunoblots of protein extracts from RAW264.7 cells that were treated with SEA (20 μg/ml) for 24 h. Representative immunoblots of three independent experiments are shown, and then protein was subjected to SDS/PAGE, immunoblotting with antibodies to AMPK, p-AMPK, ACC, p-ACC, AKT, p-AKT, mTORC1, p-mTORC1, PI3K and p-PI3K, and quantification. Band density was measured by ImageJ software and normalized to β-actin (Student’s t-test: *P < 0.05, **P < 0.01, ***P < 0.001). RAW264.7 cells were treated with SEA (20 μg/ml) with or without phosphorylated AMPK inhibitor (dorsomorphin), phosphorylated AKT inhibitor (dihydrochloride) and phosphorylated mTOR inhibitor (rapamycin) for 24 h. After that, cell culture medium was collected for measurement of glucose concentrations (**b**), and intracellular lipid accumulation of cells was determined by oil red O staining (**c**) (lipid droplets *vs* the cell section area). Ten random fields of each group were chosen for the quantification of intracellular lipids by oil red O staining by using the ImageJ software (ANOVA/LSD: *P < 0.05, **P < 0.01, ***P < 0.001)

To understand the molecular mechanisms behind the observed phenotype, we next stimulated macrophages with SEA *in vitro*. Results obtained show that SEA stimulation is associated with a comparable increase in the level of phosphorylated AMPK (Thr172) but no change in total AMPK abundance in macrophages (Fig. 4a). In addition, a similar increase in the level of phosphorylated AKT (T308) was noted in macrophages after SEA stimulation. Furthermore, mTORC1, which plays an important role in controlling the stability between anabolism and catabolism [18], was also enhanced in the phosphorylated (S2448) form but not total protein level in macrophages with SEA stimulation. However, there were no significant differences with respect to phosphorylated PI3K (Y467/Y199) or total abundance between SEA-treated and control group in this cell line (Fig. 4a).

To further clarify the roles of phosphorylated-AMPK, phosphorylated-AKT and phosphorylated-mTOR pathways on glycolipid metabolism under SEA stimulation, we used the inhibitors of phosphorylated-AMPK, phosphorylated-AKT and phosphorylated-mTORC1 to treat PBS/SEA-stimulated macrophages. Results showed that SEA stimulation decreased the glucose levels in macrophages, while suppression of phosphorylated-AMPK (Additional file 4: Figure S3a), phosphorylated-AKT (Additional file 4: Figure S3b) and phosphorylated-mTORC1 (Additional file 4: Figure S3c) in SEA-stimulated macrophages could increase glucose levels (Fig. 4b) (ANOVA and LSD multiple comparison test: *F*_(3, 11)_ = 85.96, *P* < 0.0001; *F*_(3, 11)_ = 51.71, *P* < 0.0001; *F*_(3, 11)_ = 211.3, *P* < 0.0001). In addition, SEA-stimulated macrophages showed a significant decrease in the lipid levels, while suppression of phosphorylated-AMPK, but not phosphorylated-AKT and phosphorylated-mTORC1, promoted lipid accumulation in macrophages (Fig. 4c, Additional file 4: Figure S3d) (ANOVA and LSD multiple comparison test: *F*_(7, 79)_ = 7.813, *P* < 0.0001).

### The effects of macrophages on hepatocytes glycolipid metabolism *in vitro*

**Fig. 5:**
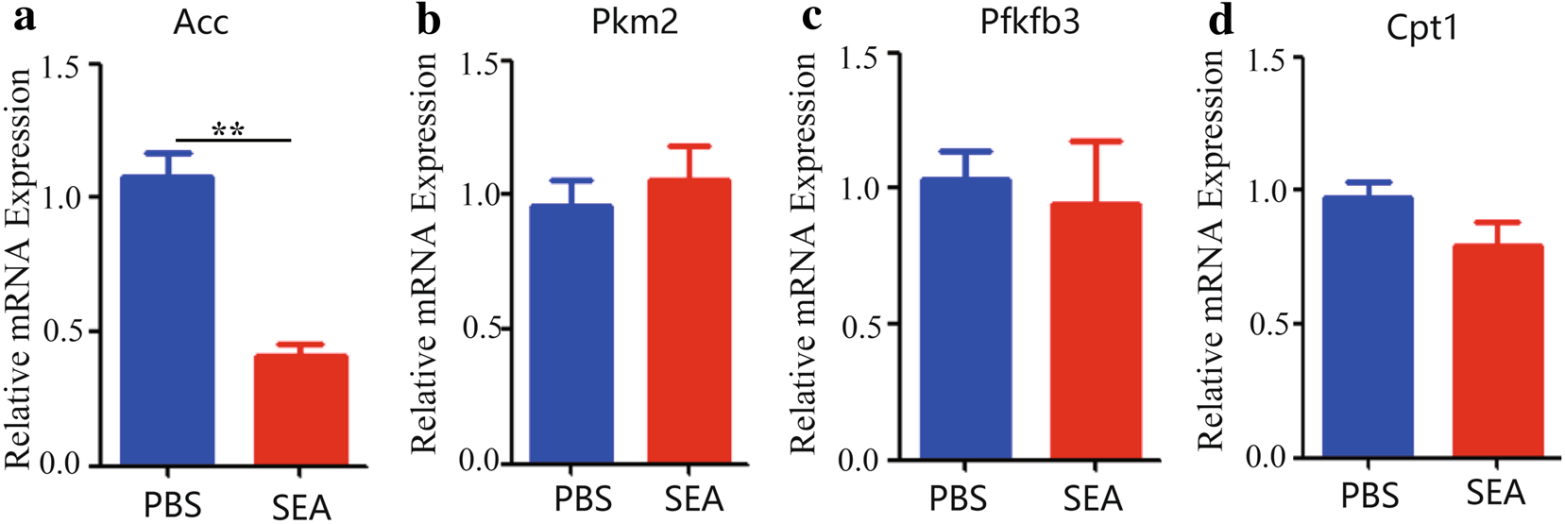
Effect of SEA-treated macrophage on the expression of genes related glycolipid metabolism in hepatocytes. **a**–**d** RAW264.7 cells were stimulated with SEA (25 μg/ml) for 24 h at 37 °C. Then, unstimulated FL83b cells were seeded in lower chamber of the transwell with 0.4 μm Pore Polycarbonate Membrane Insert and the SEA-activated RAW264.7 cells were added into upper chamber and co-cultured for another 24 h. After that, the FL83b cells were collected in the lower chamber and the expression of metabolic genes using real-time PCR was analyzed. Transcript levels for each gene in livers are expressed as fold change over transcript levels in PBS-treated control cells. Data are expressed as the mean ± SD of three independent experiments with similar results (Student’s t-test: **P < 0.01, ***P < 0.001)

Given that hepatocytes play a critical role in metabolic conversions underlying diverse physiological or pathological functions [19], we further examined the role of SEA-activated macrophages on hepatocytes metabolism. Results showed that SEA-stimulated macrophages, which showed a M2-dominant phenotype (Fig. 3c), significantly reduced the expression of Acc mRNA in hepatocytes (t-test; Acc: *t*_(4)_ = 6.869, *P* = 0.0024) (Fig. 5a); however, the SEA-activated macrophages (M2) did not affect the expression of other genes that related to metabolism in hepatocytes (t-test; Pkm2: *t*_(4)_ = 0.5789, *P* = 0.5937; Pfkfb3: *t*_(4)_ = 0.3680, *P* = 0.7315; Cpt1: *t*_(4)_ = 1.606, *P* = 0.1836) (Fig. 5b–d). Together, our results suggest that the egg antigens-activated macrophages may participate in the regulation of hepatocytes lipid metabolic gene expression.

## Discussion

Schistosome infection can lead to strong natural type 2 immune responses, and epidemiological studies have revealed that schistosome infections inversely correlate with metabolic syndrome [1, 2]. Indeed, animal studies further confirmed that chronic *S. mansoni* infection or injection with SEA improved insulin sensitivity and reduced fat mass in high diet-induced obese mice [12]. Another metabolic consequence of schistosome infection is the disturbance of glycolysis, which is characterized by significant decreases in plasma and liver glucose and glycogen, as well as the accumulation of liver lactate and urinary pyruvate [20, 21]. However, the signaling pathways that are potentially involved in the energy metabolism during schistosome infection remain unclear, and the metabolism-related gene changes that take place in the liver have not yet been characterized at the cellular level.

We previously reported that chronic exposure to *S*. *japonicum* induces a type 2 immune response in the liver and improved insulin sensitivity and glucose tolerance [5]. Here, we have further performed in-depth metabolic profiling, which showed that *S*. *japonicum* infection specifically promoted glycolysis-related gene expression, but reduced the expression of genes related to glucose synthesis in the livers of *S*. *japonicum*-infected mice. In support of these results, we also found that the level of phosphorylated of AKT, which was reported to be sufficient to accelerate glycolytic cell growth and improve metabolic parameters [22], was upregulated in the liver of mice infected with *S*. *japonicum*. These results suggested that *S*. *japonicum* eggs deposited in the liver can directly influence glucose metabolism. Interestingly, there was a striking change in glycolysis-related gene expression at 7 and 11 weeks post-infection. The reason for this may be derived from the egg production after the worm copulation 4 weeks post-infection [23], which peaks at 6–8 weeks post-schistosome-infection and persists in the liver for a long time [24]. In consistency with these observations, our *in vitro* data showed similar results, as evidenced by the increase in gene expression involved in glycolysis in SEA-stimulated macrophages.

Previous animal and proteomic studies showed the marked suppression of lipoproteins in the plasma of *S. japonicum*-infected mice [20, 25]. One explanation is that host phospholipids and triacylglycerols are taken up by adult schistosomes [26]. Another possibility is the impaired intestinal lipid absorption during schistosome infection [27]. However, our data regarding changes in the expression of genes related to fatty acid oxidation and fatty acid synthesis, as well as lipid uptake in our experimental conditions, specifically after egg production, which led us to speculate that the decreased lipoproteins in *S. japonicum-*infected mice may be partially due to abnormal lipid metabolism efficiency in the liver, which needed further study to confirm.

We further explored the potential metabolic pathways involved in the infection process. AMPK is a key cellular sensor for energy homeostasis by regulating the metabolism of glucose and lipids by promoting glycolysis and fatty acid oxidation and limiting fatty acid synthesis [28]. As an important regulator in glucose metabolism, AKT had been reported to promote glycolysis through increasing GLUT1 trafficking to the cell surface, which is involved in phosphofructokinase (PFK) and hexokinase (HK) activation [29]. Additionally, mTORC1 plays an integral part in regulating anabolism and catabolism, as defects in mTORC1 regulation results in a variety of human diseases, including type 2 diabetes [30]. Our *in vitro* data showed that *S*. *japonicum* egg antigen may exert a strong metabolic pathway effect related to AMPK, AKT and mTORC1 signaling activation in macrophage. Interestingly, by using the phosphorylated inhibitors to AMPK, AKT and mTORC1, we found that all of these signaling molecules contributed to the egg antigen-regulated glucose metabolism in macrophages. However, only the AMPK signaling molecule was found to be necessary for lipid metabolism in SEA-stimulated macrophages.

The interplay between macrophages and hepatocytes in the liver microenvironment may provide new insights into understanding the mechanism of metabolism changes [31]. During *S. japonicum* infection, macrophages were reported to typically shift from the M1 towards the M2 phenotype [32], and this process may be related to the expression of scavenger receptor-A or PPAR-γ [33, 34]. Notably, liver macrophages play a critical role in promoting lipid storage through regulating the oxidation of fatty acids in hepatocytes [35, 36], which may be related to PPARδ signaling in macrophages [37]. Our results indicate that SEA-treated macrophages, which are characterized by the M2 phenotype, play a potential role in the reduction of the fatty acid synthesis related gene (Acc) in hepatocytes.

## Conclusions

This study identified the dynamic metabolic gene expression in the liver during *S*. *japonicum* infection and illustrated egg antigen-induced cellular metabolic response through AMPK, AKT and mTORC1 signaling. Our results highlight the macrophage as a new direction for understanding aberrant glycolipid metabolism during schistosome infection.

## Additional files

**Additional file 1: Table S1.** The primer sequences used in detecting the levels of mRNA.

**Additional file 2: Figure S1.** Dynamic of the expression of genes associated with hepatic glucose metabolism in control mice.

**Additional file 3: Figure S2.** Dynamic of the expression of genes associated with hepatic lipid metabolism in control mice.

**Additional file 4: Figure S3.** The effects of inhibitors to p-AMPK, p-AKT and p-mTOR expression in macrophages.

## Data Availability

The data that support the findings of this study are included within the article and its additional files.

## Abbreviations

PI3K: phosphatidyl inositol 3 kinase
mTOR: mammalian target of rapamycin
ACC: acetyl-CoA carboxylase
AKT: protein kinase B
AMPK: AMP-activated protein kinase
GLUT: glucose transporter
PDK1: 3-phosphoinositide-dependent protein kinase 1
PFK: ATP-dependent 6-phosphofructokinase
PFKFB: 6-phosphofructo-2-kinase/fructose-2,6-biphosphatase
LDHA: lactate dehydrogenase A
PKM2: pyruvate kinase muscle isozyme
G6PC: glucose-6-phosphatase catalytic subunit
HK2: hexokinase 2
ENO3: enolase 3
ACOX1: acyl-CoA oxidase 1
CPT1: carnitine palmitoyltransferase 1
UCP2: uncoupling protein 2
ATP5B: ATP synthase subunit-β
Pparg: peroxisome proliferator activated receptor gamma
FAS: fatty acid synthase
SCD1: stearoyl-CoA desaturase 1
SREBP1C: sterol regulatory element binding protein 1c
Aldoc: fructose-bisphosphate aldolase C
