# Supplementary material for: Schistosoma japonicum infection causes a reprogramming of glycolipid metabolism in the liver
Source: Parasit Vectors. 2019 Aug 2;12:388. doi: 10.1186/s13071-019-3621-6 (PMC6679454; doi:10.1186/s13071-019-3621-6)
Supplement: Supplementary file 1 — Additional file 1: Table S1. The primer sequences used in detecting the levels of mRNA. [file 13071_2019_3621_MOESM1_ESM.pdf]

Additional file 1: Table S1. The primer sequences used in detecting the levels of mRNA

**Gene name**	**Gene ID**	**Forward (5'—3')**	**Reverse (5'—3')**
Glut1	20525	GCAGTTCGGCTATAACACTGG	GCGGTGGTTCCATGTTTGATTG
Pdk1	228026	GGATGTATAATCTATCAGCTCGT	TTCCACAAGATCTCTAGCCTT
Pkm2	18746	GGAGCCACTCTGAAGATCACC	ACTTCTCCATGTAAGCGTTGTCC
G6pc	14377	CTACTCTTGCTATCTTTCGAGGA	AACACCGGAATCCATACGTT
Hk2	15277	ATCTACGCCATTCCCGAGGACA	ATCTCTGCCTTCCACGCCACT
Pfkfb3	170768	AAGAAGCTGACTCGCTACCTC	AGTTGTACTCATTCTCGCCAT
Aldoc	11676	TACCGCCAGGTCCTATTCAGT	CCCAGCTAGAGGCACTACACC
Eno3	13808	TCTCTACCGACACATCGCAGA	GCTACATCCATGCCGATCACC
Pfk	18641	GAGCAGCCTCCATACCACA	CTCGAGCTGGTAAACCCAC
Ldha	16828	ATGGCAACCCTCAAGGACCAG	CATGCCAACAGCACCAACCC
Scd1	20249	CTCTACACCTGCCTCTTCGG	GCCGTGCCTTGTAAGTTCTG
Srebp1c	20787	CAGAGCCGTGGTGAGAAGC	GCAAGAAGCGGATGTAGTCG
Acc	107476	CACCAGTTTTGCATTGAGAA	TACGCTGTTGAGTTCATAGGCC
Fas	14104	AGGTGGTGATAGCCGGTATG	TGGGTAATCCATAGAGCCCAGT
PPARg	19016	AGAACCTGCATCTCCACCTT	CCCACAGACTCGGCACTCAATA
Cpt1	12894	GTGTCCAAGTATCTGGCAGT	CAGGGTATTTCTCAAAGTCAAC
CD36	20778	TGGTCAAGCCAGCTAGAAA	TCCCAAGTAAGGCCATCTC
Acox1	11430	ATGCCTTTGTTGTCCCTATCCG	CTGAGCTGCACTTCCCACGAG
Atp5b	11947	ATCAACAATGTCGCCAAAGCC	ATTCAGCAACGGTCAAACCAG
Ucp2	22228	CCTCTGGATACCGCCAAGGTC	TGACTCTCCCCTTGGATCTGC
β-actin	11461	GTGACGTTGACATCCGTAAAGA	GCCGGACTCATCGTACTCC
