# Supplementary material for: Schistosoma japonicum infection causes a reprogramming of glycolipid metabolism in the liver
Source: Parasit Vectors. 2019 Aug 2;12:388. doi: 10.1186/s13071-019-3621-6 (PMC6679454; doi:10.1186/s13071-019-3621-6)
Supplement: Supplementary file 2 — Additional file 2: Figure S1. Dynamic of the expression of genes associated with hepatic glucose metabolism in control mice. [file 13071_2019_3621_MOESM2_ESM.pdf]

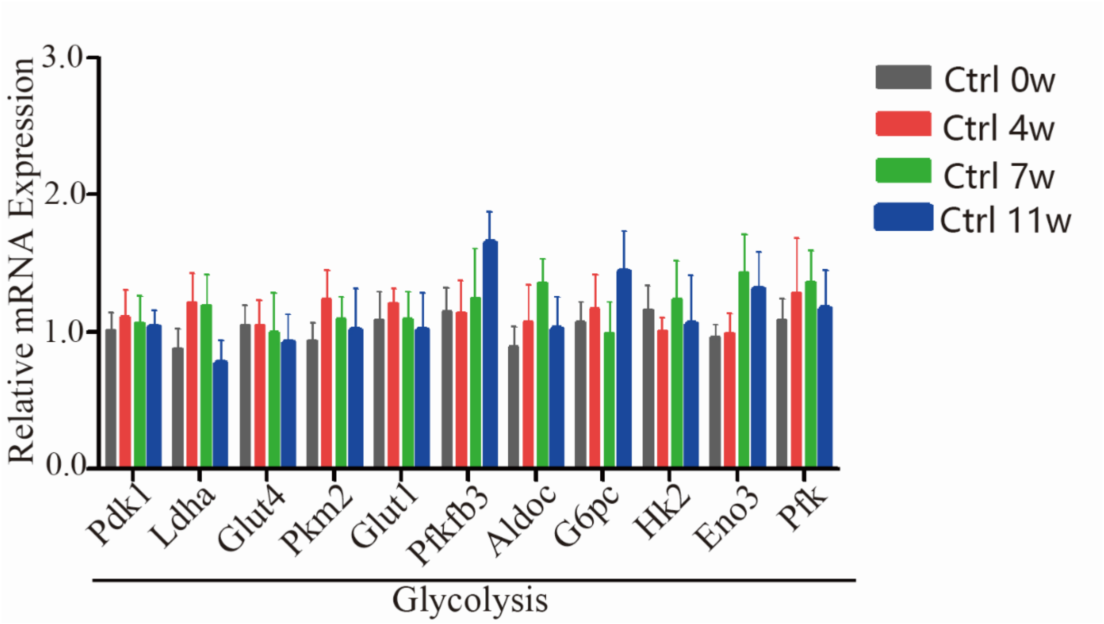

Additional file 2: Fig. S1. Dynamic of the expression of genes associated with hepatic glucose metabolism in control mice

Liver tissues were prepared from control mice at the time points (0, 4, 7, and 11 weeks post-infection). The mRNA levels of glycolysis-related important genes (Pdk1, Ldha, Glut4, Pkm2, Glut1, Pfkfb3, Aldoc, HK2, Eno3, Pfk) and gluconeogenesis gene (G6pc) in the liver of each group were evaluated by real-time PCR. The mRNA level of each gene was normalized to β-actin mRNA levels in each sample (ANOVA/LSD).
