# Supplementary material for: Schistosoma japonicum infection causes a reprogramming of glycolipid metabolism in the liver
Source: Parasit Vectors. 2019 Aug 2;12:388. doi: 10.1186/s13071-019-3621-6 (PMC6679454; doi:10.1186/s13071-019-3621-6)
Supplement: Supplementary file 3 — Additional file 3: Figure S2. Dynamic of the expression of genes associated with hepatic lipid metabolism in control mice. [file 13071_2019_3621_MOESM3_ESM.pdf]

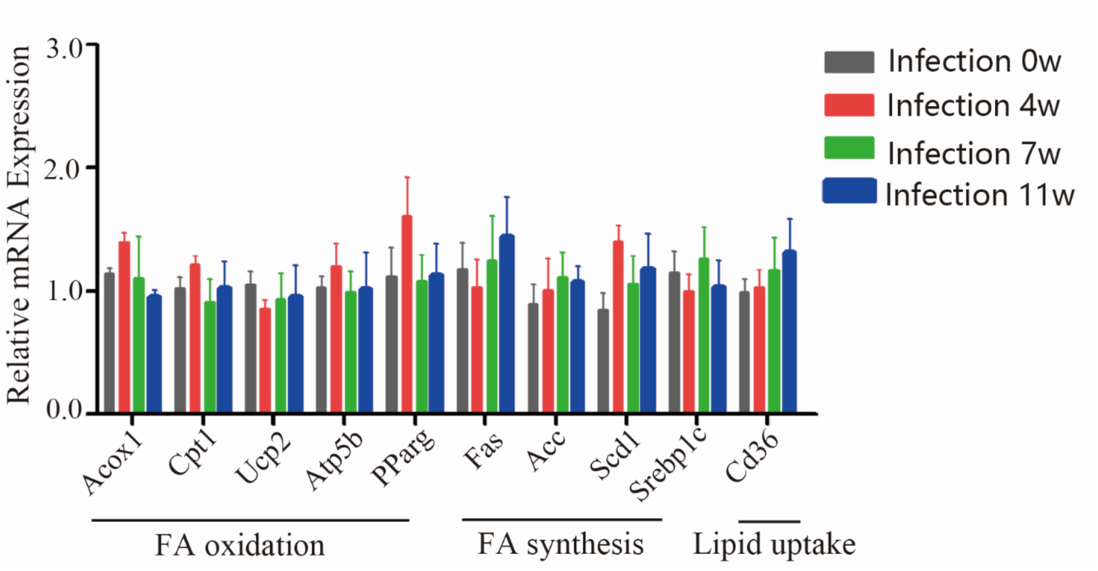

Additional file 3: Fig. S2. Dynamic of the expression of genes associated with hepatic lipid metabolism in control mice

Liver tissues were prepared from control mice at the time points (0, 4, 7, and 11 weeks post-infection). Expression of fatty acid (FA) oxidative-related genes (Acox1, Cpt1, Ucp2, Atp5b, Pparg), the FA synthesis genes (Fas, Acc, Scd1, Srebp1c) and lipid uptake gene (CD36) in the liver was evaluated by real-time PCR. The mRNA level of each gene was normalized to β-actin mRNA levels in each sample (ANOVA/LSD).
