# Supplementary material for: Schistosoma japonicum infection causes a reprogramming of glycolipid metabolism in the liver
Source: Parasit Vectors. 2019 Aug 2;12:388. doi: 10.1186/s13071-019-3621-6 (PMC6679454; doi:10.1186/s13071-019-3621-6)
Supplement: Supplementary file 4 — Additional file 4: Figure S3. The effects of inhibitors to p-AMPK, p-AKT and p-mTOR expression in macrophages. [file 13071_2019_3621_MOESM4_ESM.pdf]

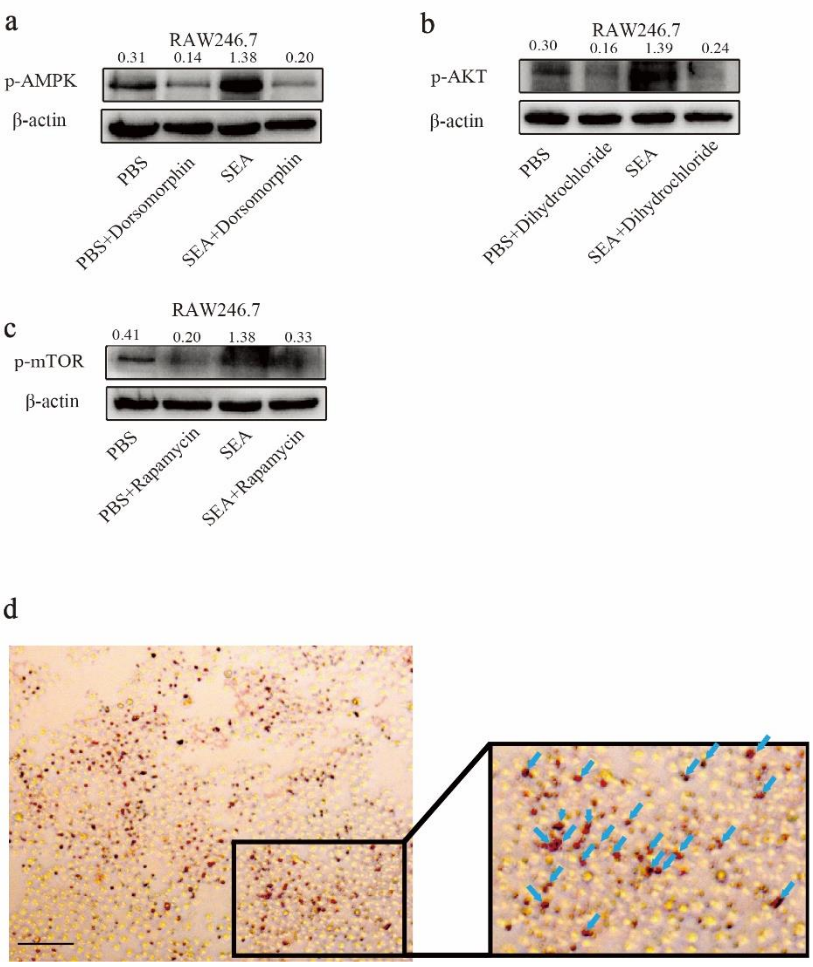

Additional file 4: Fig. S3. The effects of inhibitors to pho-AMPK, pho-AKT, and pho-mTOR expression in macrophages

Representative Western blots of pho-AMPK, pho-AKT, pho-mTOR in RAW264.7cells (**a-c**) that were treated with SEA (20 μg/ml) with or without phosphorylated AMPK inhibitor (Dorsomorphin), phosphorylated AKT inhibitor (Dihydrochloride), and phosphorylated mTOR inhibitor (Rapamycin) for 24 h. Band density was measured by ImageJ software and normalized to β-actin. **(d)** The representative figure related Figure 4c for the oil red O staining of FL83B and RAW264.7 cells that were treated with SEA (20 μg/ml) with or without phosphorylated AMPK inhibitor (Dorsomorphin), phosphorylated AKT inhibitor (Dihydrochloride), and phosphorylated mTOR inhibitor (Rapamycin) for 24 h.
